# Voltage Gated Calcium Channel Activation by Backpropagating Action Potentials Downregulates NMDAR Function

**DOI:** 10.3389/fncel.2018.00109

**Published:** 2018-04-23

**Authors:** Anne-Kathrin Theis, Balázs Rózsa, Gergely Katona, Dietmar Schmitz, Friedrich W. Johenning

**Affiliations:** ^1^Neuroscience Research Center, Charité Universitätsmedizin Berlin, Berlin, Germany; ^2^Laboratory of 3D Functional Network and Dendritic Imaging, Institute of Experimental Medicine, Hungarian Academy of Sciences, Budapest, Hungary; ^3^Faculty of Information Technology and Bionics, Pázmány Péter University, Budapest, Hungary; ^4^Einstein Center for Neuroscience, Berlin, Germany; ^5^Bernstein Center for Computational Neuroscience, Berlin, Germany; ^6^Cluster of Excellence “Neurocure”, Berlin, Germany; ^7^DZNE-German Center for Neurodegenerative Disease, Berlin, Germany; ^8^Berlin Institute of Health (BIH), Berlin, Germany

**Keywords:** dendritic spines, two-photon microscopy, calcium, synaptic transmission, voltage gated Ca^2+^ channels (VGCCs), metaplasticity, homeostatic synaptic plasticity, NMDAR

## Abstract

The majority of excitatory synapses are located on dendritic spines of cortical glutamatergic neurons. In spines, compartmentalized Ca^2+^ signals transduce electrical activity into specific long-term biochemical and structural changes. Action potentials (APs) propagate back into the dendritic tree and activate voltage gated Ca^2+^ channels (VGCCs). For spines, this global mode of spine Ca^2+^ signaling is a direct biochemical feedback of suprathreshold neuronal activity. We previously demonstrated that backpropagating action potentials (bAPs) result in long-term enhancement of spine VGCCs. This activity-dependent VGCC plasticity results in a large interspine variability of VGCC Ca^2+^ influx. Here, we investigate how spine VGCCs affect glutamatergic synaptic transmission. We combined electrophysiology, two-photon Ca^2+^ imaging and two-photon glutamate uncaging in acute brain slices from rats. T- and R-type VGCCs were the dominant depolarization-associated Ca^2+^conductances in dendritic spines of excitatory layer 2 neurons and do not affect synaptic excitatory postsynaptic potentials (EPSPs) measured at the soma. Using two-photon glutamate uncaging, we compared the properties of glutamatergic synapses of single spines that express different levels of VGCCs. While VGCCs contributed to EPSP mediated Ca^2+^ influx, the amount of EPSP mediated Ca^2+^ influx is not determined by spine VGCC expression. On a longer timescale, the activation of VGCCs by bAP bursts results in downregulation of spine NMDAR function.

## Introduction

The dendritic surface of most excitatory projection neurons is covered with thousands of spines which receive over 90% of glutamatergic synapses (Harris and Kater, 1994). Spines and the corresponding synapses are plastic, they undergo activity-dependent functional state changes. This includes spine- and synapse specific structural, morphological and functional changes ultimately affecting synapse strength and stability (Alvarez and Sabatini, 2007; Korte and Schmitz, 2016; Segal, 2017). An important signaling molecule related to different types of spine plasticity is calcium (Ca^2+^). Ca^2+^ serves as a transducer between fast and transient electrical signals at the membrane and biochemical and structural changes outlasting the initial electrical signal (Hille, 2001). Therefore, spine Ca^2+^ signals evoked by neuronal activity substantially determine plasticity processes.

Voltage gated Ca^2+^ channels (VGCCs) contribute to spine Ca^2+^ signals in synaptically excited spines. Theory predicts that spine VGCCs could actively enhance synaptic excitatory postsynaptic potentials (EPSPs; Miller et al., 1985; Segev and Rall, 1988; Araya, 2014). In a number of experimental studies, the contribution of VGCCs to synaptic depolarization during EPSPs has been described as marginal (Bloodgood and Sabatini, 2007; Palmer and Stuart, 2009; Popovic et al., 2015). In CA1 pyramidal neurons, they are part of a negative feedback loop that dampens synaptic EPSPs (Bloodgood and Sabatini, 2007; Giessel and Sabatini, 2011; Wang et al., 2014, 2015).

In addition to specific direct synaptic activation of spines, VGCCs are also activated globally in spines that are activated by electrotonic spread of depolarization and not by direct synaptic inputs (Higley and Sabatini, 2008). Whenever suprathreshold synaptic activation evokes an action potential (AP), the depolarization travels back into the dendrite in the form of backpropagating action potentials (bAPs). bAPs activate VGCCs in dendrites and spines. The resulting global Ca^2+^ signals transmit neuronal activity levels to a large population of spines that do not receive direct synaptic activation (Waters et al., 2005). Recently, we observed that neuronal AP firing upregulates spine VGCCs. In these experiments, we also observed a larger range of single spine VGCC responses to bAPs in excitatory layer 2 neurons of the MEC when directly compared to CA1 pyramidal neurons (Johenning et al., 2015). The plasticity and interspine variability of VGCC expression has not been taken into account by previous studies examining the acute effect of spine VGCCs on synaptic transmission. In addition to acute effects resulting from direct electrical interactions in spines with a high density of VGCCs, VGCC expression levels may also interfere with the long-term regulation of synaptic strength. We therefore tested if synaptic properties of spines with large depolarization-mediated VGCC Ca^2+^ transients differ from spines with small Ca^2+^ transients. AMPAR mediated synaptic transmission appeared functionally uncoupled from VGCC-mediated Ca^2+^ influx and VGCCs do not electrically amplify spine EPSPs. However, we found a reduction of the spine-specific NMDAR-response by bAP-Ca^2+^ transients. This establishes a functional link between global VGCC activation by dendritic backpropagation of APs and synaptic transmission.

## Materials and Methods

### Slice Preparation and Electrophysiology

Acute brain slices were prepared from Wistar rats (postnatal day 17–25, see Supplementary Figure S5 for age distributions in different experiments) in accordance with the national and institutional guidelines as described in Beed et al. (2010). All procedures were approved by the local health authority and the local ethics committee (Landesamt für Gesundheit und Soziales, Berlin; animal license number T0073/04). Briefly, brains were placed in ice-cold artificial cerebrospinal fluid (ACSF; pH 7.4) containing (in mM): 87 NaCl, 26 NaHCO_3_, 25 Glucose, 2.5 KCl, 7 MgCl_2_, 1.25 NaH_2_PO_4_, 0.5 CaCl_2_ and 75 Sucrose. Slices were cut at 300 μm thickness, and incubated at 35°C for 30 min. The slices were then transferred to standard ACSF containing (in mM): 119 NaCl, 26 NaHCO_3_, 10 Glucose, 2.5 KCl, 2.5 CaCl_2_, 1.3 MgCl_2_, and 1 NaH_2_PO_4_. The slices were stored at room temperature in a submerged chamber for 0.5–5 h before being transferred to the recording chamber. One micromolar of Gabazine was added for all experiments involving synaptic stimulation. For application of Ni^2+^ and the interleaved control experiments, NaH_2_PO_4_ was omitted from the standard ACSF. Application of peptide toxins (SNX-482, CtxGIVA, AgaIVA) and the interleaved control experiments were performed in a perfusion system where tubing was coated with 0.1 mg/ml cytochrome added to the standard ACSF. TTA-P2, Nimodipine, PD-173212, SKF-96365 and D-APV were added to the standard ACSF after baseline recordings. For voltage clamp experiments in Figure 4, 1 μM TTX and 10 μM D-Serine was added to the bath. In experiments shown in Figure 5, we wanted to isolate NMDAR EPSCs at hyperpolarized potentials. In these experiments, slices were recorded in low-Mg^2+^ ACSF consisting of (in mM): 119 NaCl, 26 NaHCO_3_, 10 Glucose, 2.5 KCl, 2.5 CaCl_2_, 0.1 MgCl_2_, 1 NaH_2_PO_4_, 1 μM Gabazine and 20 μM NBQX.

**Figure 1:**
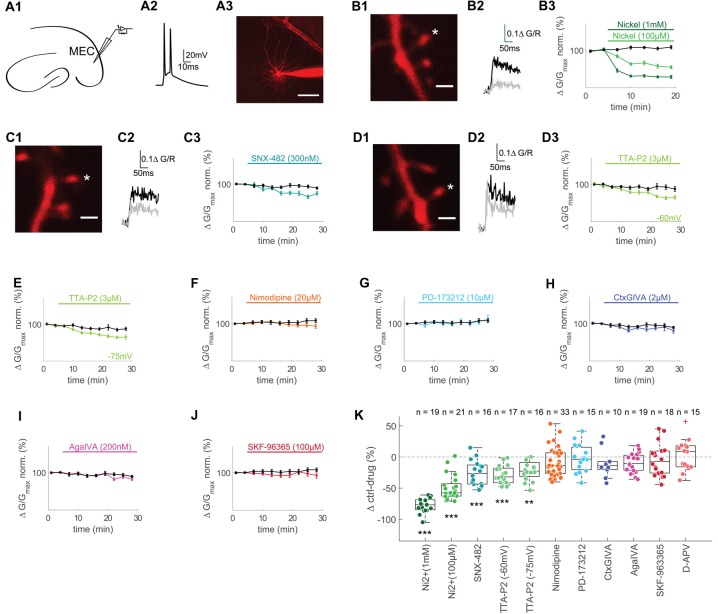
T- and R-type voltage gated Ca^2+^ channels (VGCCs) contribute to backpropagating action potentials (bAPs)-Ca^2+^ transients in spines of layer II MEC cells.** (A1)** Illustration of recording pipette positioning in layer 2 of the MEC. **(A2)** Representative AP doublet (100 Hz) evoked by current injection to induce the bAP-Ca^2+^ transients. **(A3)** Representative MEC layer 2 cell with recording pipette. Scale bar: 50 μm. **(B1)** Z-projection of the imaged spine (marked with an asterisk). Scale bar: 2 μm. **(B2)** Averaged fluorescence traces at baseline (0–5 min, black) and 10–15 min (gray) after wash-in of 1 mM Ni^2+^. **(B3)** Time plot of normalized, binned (3 min) doublet evoked bAP-Ca^2+^ transients under control conditions (black) and wash-in of 1 mM (dark green) or 100 μM Ni^2+^ (lightgreen) after 5 min of baseline. **(C1,D1)** Z-projections of the imaged spines (marked with asterisks). Scale bars: 2 μm. **(C2,D2)** Averaged fluorescence traces 0–5 min (black) and 20–25 min (gray) after wash-in of 300 nM SNX-482 **(C2)** and 3 μM TTA-P2 **(D2)**. **(C3,D3,E–J)** Time plots of normalized, binned (3 min) doublet evoked bAP-Ca^2+^ transients under control conditions (black) and wash-in of 300 nM SNX-482 **(C3)**, 3 μM TTA-P2 at −60 mV **(D3)**, 3 μM TTA-P2 at −75 mV **(E)**, 20 μM Nimodipine **(F)**, 10 μM PD-173212 **(G)**, 2 μM CtxGIVA **(H)**, 200 nM AgaIVA **(I)** and 100 μM SKF-96365 **(J)** after 5 min of baseline. **(K)** Median box plot illustrating the contribution of T- and R-type (1 mM Ni^2+^ and 100 μM Ni^2+^), R-type (SNX-482), T- type (TTA-P2 −60 mV and −75 mV), L-type (Nimodipine), N-type (PD-173212 and CtxGIVA), P/Q-type (AgaIVA), TRPC calcium channels and NMDARs to doublet evoked bAP-Ca^2+^ transients measured as Δ of the averaged time-matched interleaved control value and the drug effect 10–15 min after Ni^2+^ wash-in and 20–25 min after wash-in of all other drugs, **p < 0.01, ***p < 0.001. The red cross in the D-APV group illustrates an outlier that was included in the statistics but omitted from the figure for clarity (90.2 Δ control-drug (%)).

**Figure 2:**
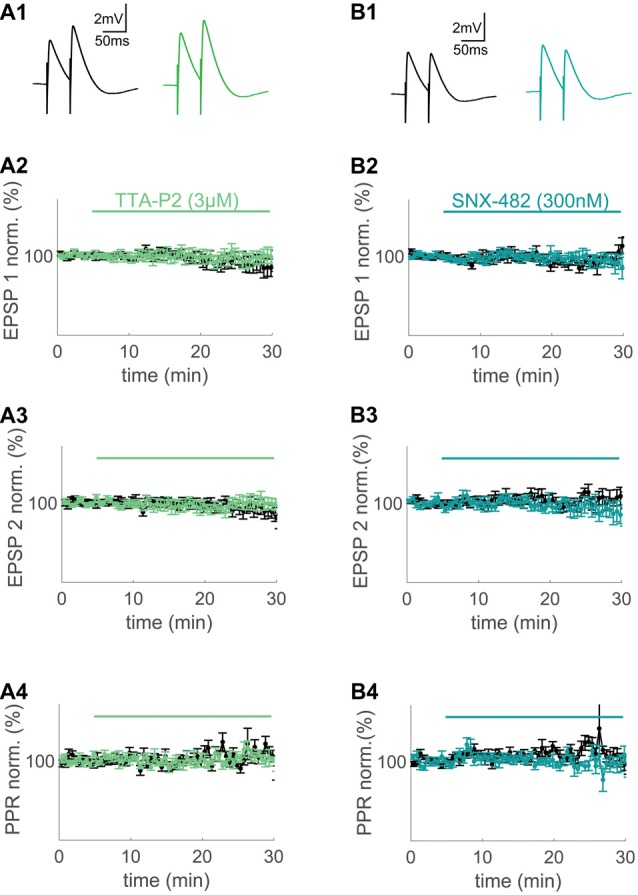
T- and R-type VGCCs do not contribute to synaptically evoked compound excitatory postsynaptic potentials (EPSPs) measured at the soma.** (A1,B1)** Extracellular synaptic paired-pulse stimulation evoked compound EPSPs under control conditions (black) and after wash-in of 3 μM TTA-P2 (green) and 300 nM SNX-482 (blue) 20–25 min after wash-in. **(A2,A3,B2,B3)** Time plots of normalized, binned (1 min) maximum amplitudes of EPSP1 **(A2,B2)** and EPSP2 **(A3,B3)** under control conditions (black) and during wash-in of TTA-P2 (green) and SNX-482 (blue). **(A4,B4)** Paired-pulse ratio (PPR) of the control conditions (black) and during wash-in of TTA-P2 (green) and SNX-482 (blue).

**Figure 3:**
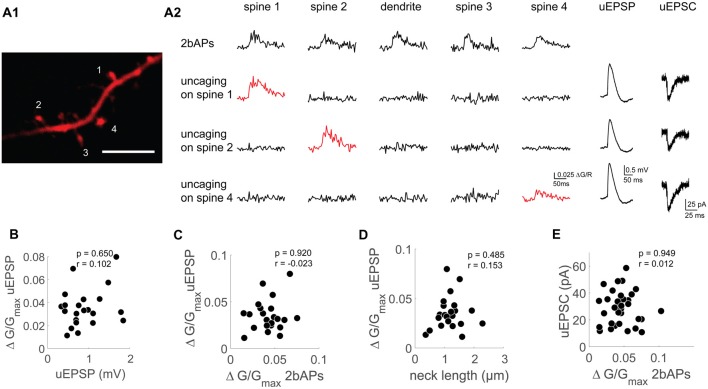
Functional uncoupling between bAP Ca^2+^ transients, uEPSP mediated Ca^2+^ transients and AMPAR-mediated uEPSCs on the single spine level. **(A1)** Z-projection of the imaged dendritic segment. Scale bar: 2 μm. **(A2)** Doublet evoked bAP-Ca^2+^ transients (2bAPs), uncaging evoked Ca^2+^ transients with corresponding uEPSPs and uEPSCs, spines are marked in **(A1)**. Doublet evoked bAP-Ca^2+^ transients and uncaging evoked Ca^2+^ transients were also recorded in the adjacent dendritic segment. **(B)** Scatter plot of uEPSPs vs. uncaging evoked Ca^2+^ transients. Statistics: spearman correlation. **(C)** Scatter plot of doublet evoked Ca^2+^ transients and uncaging evoked Ca^2+^ transients. Statistics: spearman correlation. **(D)** Correlation of neck length and uncaging evoked Ca^2+^ transients. Statistics: spearman correlation. **(E)** Correlation of doublet evoked Ca^2+^ transients and uEPSCs. Statistics: spearman correlation.

**Figure 4:**
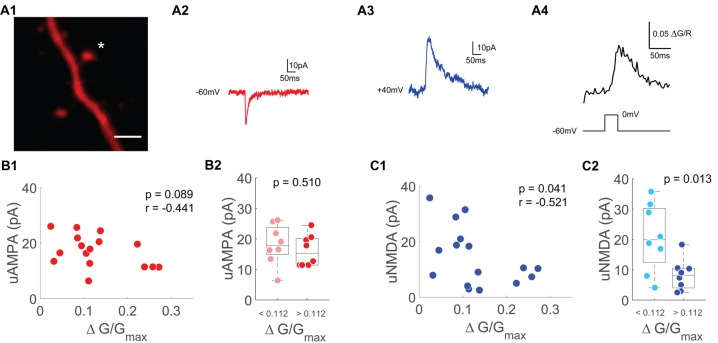
Single spine uNMDA EPSCs are negatively correlated with depolarization induced spine Ca^2+^ transients.** (A1)** Z-projection of the imaged spine (marked with an asterisk). Scale bar: 2 μm.** (A2)** Averaged example trace of an uncaging evoked current at −60 mV. **(A3)** Averaged example trace of an uncaging evoked current at +40 mV. **(A4)** Averaged Ca^2+^ transients evoked by a depolarization step from −60 mV to 0 mV for 30 ms. **(B1)** Scatter plot of depolarization step evoked Ca^2+^ transients and uncaging evoked currents at −60 mV (uAMPA). Statistics: spearman correlation. **(B2)** Median box plot of uAMPA-EPSC amplitudes of spines with smaller (light red) and larger (red) depolarization mediated VGCC Ca^2+^ transients grouped by the median. Statistics: *t*-test. **(C1)** Scatter plot of depolarization step evoked Ca^2+^ transients and uncaging evoked currents at +40 mV (uNMDA) measured 50–60 ms after onset. Statistics: spearman correlation. **(C2)** Median box plot of uNMDA-EPSC amplitudes of spines with smaller (light blue) and larger (blue) depolarization mediated VGCC Ca^2+^ transients grouped by the median. Statistics: *t*-test.

**Figure 5:**
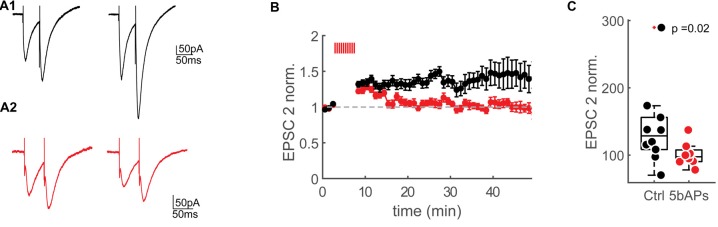
Reduction of NMDAR-currents by AP firing.** (A1)** Extracellular synaptic paired-pluse stimulation evoked EPSCs under control conditions at baseline (0–2 min) and 30–35 min after the control window (stimulation artifacts were clipped). **(A2)** Extracellular synaptic paired-pulse stimulation evoked EPSCs at baseline (0–2 min) and 30–35 min after bursts of 5bAPs (stimulation artifacts were clipped). **(B)** Time plots of normalized, binned (1 min) maximum amplitudes of the second EPSC (EPSC2) under control conditions (black) and after applying 10 bursts of 5bAPs (indicated with red lines, red, plotted as mean ± SEM). **(C)** Median box plot of the normalized EPSC2 amplitudes of the control experiments and after 10 bursts of 5bAPs) time point: 30–35 min after induction or control window). Statistics: Wilcoxon-Mann-Whitney Test.

Whole-cell current clamp experiments were performed at near physiological temperature (32–34°C) using an Axon Multiclamp 700B amplifier (Molecular Devices, Sunnydale, CA, USA). Signals were low pass filtered at 2 kHz and digitized at a sampling rate of 5 kHz (BNC-2090, National Instruments Corporation, Austin, TX, USA). For experiments involving current clamp measurements or AP firing, pipettes (3–6 MΩ) were filled with an intracellular solution containing (in mM): 130 K-gluconate, 20 KCl, 10 HEPES, 4 Mg-ATP, 0.3 Na-GTP and 10 phosphocreatine (pH: 7.3). Synaptic stimulation in Figure 2 was performed using an intracellular solution containing (in mM): 130 KMeSO_3_, 10 KCl, 10 HEPES, 4 NaCl, 4 Mg-ATP, 5 phosphocreatine, 0.5 Na-GTP. For voltage clamp experiments in Figure 4, the intracellular solution consisted of (in mM): 135 Cesium-Methansulfonate, 10 Hepes, 10 Phosphocreatine, 4 NaCl, 4 Mg-ATP and 0.4 Na-GTP. For 2P-imaging, 30 μM Alexa-594 and 200 μM Fluo-4FF (exps. in Figure 3) or 500 μM Fluo-5F (exps. in Figure 1, Supplementary Figure S4 and Figure 4) were added. For 2P-uncaging, 2.5 mM of DNI caged glutamate was used in a closed perfusion circuit with a total volume of 20 ml. To keep the osmolarity constant, evaporation was compensated by constantly adding ddH_2_O using a Heparin perfusor. Under these conditions, fluctuations of osmolarity <10% were confirmed in test experiments. Initial series resistances were between 6 MΩ and 20 MΩ. APs were induced with 2 ms square current pulses ranging from 1 to 3 nA. Doublets and quintuplets were delivered at 100 Hz. Experiments were aborted if the holding current exceeded −200 pA at −60 mV. See Supplementary Table S1 for a synopsis of holding currents and resting membrane potentials of all experiments. Liquid junction potential (LJP) was not corrected. Synaptic stimulation was performed using an extracellular stimulation electrode. Bridge balance compensation was applied in current clamp, and series resistance in voltage clamp was not allowed to increase by more than 20%.

### Two-Photon Calcium Imaging and Uncaging

A Femto 2D two-photon laser scanning system (Femtonics Ltd., Budapest, Hungary) was equipped with two femtosecond pulsed Ti:Sapphire lasers (Cameleon, Coherent, Santa Clara, CA, USA). The imaging laser was tuned to *λ* = 805 nm for Alexa-594 (red fluorescence (R), morphometric dye) and Fluo-4FF or Fluo-5F (green fluorescence (G) low and high affinity Ca^2+^ indicators, respectively). The uncaging laser was tuned to 720 nm. The laser lines were directly coupled into the microscope, precise overlay of the imaging and uncaging laser lines was checked directly before starting an experiment using fluorescent spheres. Imaging and uncaging were controlled by the Matlab-based MES software package (Femtonics Ltd., Budapest, Hungary). For delivery of uncaging pulses and detection of fluorescence we used a water immersion objective (LUMPLFL 60×/1.0 NA, Olympus, Hamburg, Germany). Transfluorescence and transmitted infra-red light were detected using an oil immersion condenser (Olympus).

We filled the cells for at least 25 min with dye before multiple line-scans of dendritic spines and the adjacent dendritic segment were taken. Spines were within 150 μm of the soma to reduce space-clamp errors. Imaged spines were located up to 75 μm under the slice surface. The average scanning speed was 300 Hz and the intermediate sections were jumped over within 60 μs using a spline interpolated path (Lörincz et al., 2007). bAP-Ca^2+^ transients of the doublet test stimulus were measured every 60 s. uEPSP Ca^2+^ transients where measured at 300 Hz by interleaving the uncaging point and the imaging line in the multiple line scan mode applying the aforementioned spline interpolated path (Bywalez et al., 2015). 0.5–1 ms duration uncaging pulses were generated using an electro-optical modulator (Pockels cell, Conoptics). Energy deposition of the uncaging laser for each individual spine of interest was calibrated by placing a fluorescent calibration microsphere from the same batch (InSpeck Green, Thermo Fischer Scientifics) in a sealed patch pipette in the focal plane next to the spine. Laser power was then set to always reach the same absolute brightness value under identical detection settings (Supplementary Figure S3). Based on the minuscule intrabatch variability of the beads, we assumed identical inter-slice and inter-animal energy deposition of the uncaging laser. At a distance of about 0.5 μm from the spine head, we then searched for the maximal activation point of each spine. Before each uncaging sweep, the position of the spinehead and the uncaging point were realigned to compensate for movements. Care was taken to only image isolated spines >2 μm from a neighboring spine in order to minimize cross-activation of different spines.

Calibration of G_max_/R values (ratio of maximal green fluorescence change under saturating [Ca^2+^] over red) was performed at the tip of a sealed pipette in the imaging plane of the slice using a calibration solution consisting of 50 μl recording solution and 50 μl 1 M CaCl_2_ for each batch of recording solution. For statistical comparisons and display of averages from different experiments (Supplementary Figure S4), changes in [Ca^2+^] are reported as G/G_max_ obtained by dividing ΔG/R values by their corresponding G_max_/R values (Holderith et al., 2012). Typical traces from single experiments are averages of 2–6 sweeps and displayed as ΔG/R values.

For quantification of bAP-Ca^2+^ amplitudes, G/G_max_ was averaged over a 70 ms time interval starting 10 ms after the first AP. uEPSP Ca^2+^ amplitudes were averaged over a 20 ms time interval starting 20 ms after the uncaging pulse. The depolarization pulse in voltage clamp was averaged over a 30 ms time interval. The analysis intervals are based on visual inspection of the data for maximizing the signal to noise ratio. ΔG/G_max_ amplitudes are averages of 2–6 sweeps.

To eliminate out of focus line scan measurements, measurements were excluded when the Alexa-594 intensity was below 80% of the average baseline intensity. A further exclusion criterion was the rise in the background corrected baseline green over red (G_0b_R) above 50% of the average baseline value in three consecutive sweeps. Increases in G_0b_R indicate a rise in baseline Ca^2+^ suggesting a deterioration in cell health (Yasuda et al., 2004).

In experiments where relative changes of the bAP-Ca^2+^ transient were measured (Figure 1), spines were only included if at least two out of six sweeps in the baseline and analysis time window fulfilled the above mentioned criteria for focus and cell health. In addition, in order to form a meaningful post/pre percentage ratio, we defined an inclusion criterion for the signal-to-noise ratio (S/N) of the pre-induction signal: in averaged traces of the pre-induction sweeps, the amplitude averaged in the 20 ms time interval 10 ms after the second AP in a doublet had to be 2.5 times larger than the standard deviation of a 40 ms pre-AP baseline stretch.

For morphological reconstructions, we performed *post hoc* high-resolution z-stacks of the recorded spines with a Δz of 0.2 μm. Head size and spine length were estimated from maximum intensity projections of z-stacks of the spines and the adjacent dendritic segment. The apparent spine size was approximated by measuring the FWHM of the maximal spine diameter x. The diameter of spines is below the resolution limit of a two-photon microscope. We therefore implemented a correction factor k by dividing the maximal brightness of a spine by the maximal brightness of the adjacent dendritic segment. This correction is based on the assumption that the dendritic segment is larger than the resolution limit of the 2P microscope (Holtmaat et al., 2005). Spine length was determined from the origin of the spine at the dendrite to the middle of the spine head.

### Statistics

For statistical comparisons, we used GraphPad Prism Software^1^ or the MATLAB Statistics toolbox. Datasets were tested for normal distribution using a Shapiro-Wilk normality test. In case of non-normally distributed data, the non-parametric two-tailed Wilcoxon-Mann-Whitney U test was used unless otherwise noted. When data was distributed normally, two-tailed *t*-tests were used as indicated. Correlations were tested using the non-parametric Spearman’s rank order test unless otherwise noted. Significance level for all statistical tests was at *p* < 0.05. Unless otherwise noted, data are reported as median ± interquartile range.

## Results

### VGCC Content of MEC Layer 2 Spines Measured With bAPs

The bAP-Ca^2+^ transient is mainly set by the VGCC channel conductances (Bloodgood and Sabatini, 2007). We can therefore define the bAP-Ca^2+^ transient as a proxy for the spine VGCC content. In acute brain slices, we studied excitatory neurons in layer 2 of the medial entorhinal cortex (MEC) in the whole-cell patch clamp configuration. We performed two-photon Ca^2+^ imaging of bAP-Ca^2+^ transients in spines using Fluo-5F (500 μM) as a Ca^2+^ indicator and Alexa 594 (30 μM) as a morphometric dye (Figure 1A). We analyzed the contribution of different subtypes of VGCCs to doublet bAP-Ca^2+^ transients using different antagonists. For these experiments, we chose a relatively high concentration of a medium-affinity Ca^2+^ indicator in order to improve our signal to noise ratio and suppress VGCC plasticity (Johenning et al., 2015). Every 60 s, we imaged doublet bAP-Ca^2+^ transients evoked by current injection. The effect of drug-wash-in was normalized to the 5 min pre-wash-in baseline. Drug effects were compared to the averaged control experiments (ctrl) interleaved with the drug application experiments under similar recording conditions (time-matched controls) and expressed as Δctrl-drug in Figure 1K.

At a concentration of 100 μM, the divalent cation Ni^2+^ partially blocks both R- and T-type VGCCs (Randall and Tsien, 1995; Foehring et al., 2000). This resulted in a significant reduction of spine bAP-Ca^2+^ transients measured at a time interval 10–15 min after Ni^2+^ wash-in compared to time-matched controls (100 μM Ni^2+^: *n* = 21/6/3 (spines/cells/animals), ctrl: *n* = 22/5/3 (spines/cells/animals), Ni^2+^ vs. ctrl: *p* < 0.0001 (*t*-test), Δctrl-Ni^2+^ = −57.6 ± 19.9%, Figures 1B,K). When using 1 mM Ni^2+^, complete block of R- and T-Type channels further reduced spine bAP-Ca^2+^ transients at a time interval 10–15 min after Ni^2+^ wash-in (1 mM Ni^2+^: *n* = 19/5/4 (spines/cells/animals), ctrl: *n* = 22/5/3 (spines/cells/animals), Ni^2+^ vs. ctrl: *p* < 0.0001 (*t*-test), Δctrl-Ni^2+^ = −76.9 ± 16.1%, Figures 1B,K).

Given the poor selectivity of Ni^2+^, we wanted to use more specific drugs to antagonize T- and R-Type VGCCs. When we applied the R-type VGCC blocker SNX-482 (300 nM), we reached a significant reduction of the spine bAP-Ca^2+^ transient between 20 and 25 min after wash-in when compared to time-matched controls (SNX-482: *n* = 16/5/4 (spines/cells/animals), ctrl: *n* = 37/8/6 (spines/cells/animals), SNX-482 vs. ctrl: *p* = 0.0003, Δctrl-SNX-482 = −26.5 ± 30.6%, Figures 1C,K). To selectively inhibit T-Type VGCCs, we used TTA-P2, an antagonist extensively characterized in thalamocortical relay neurons (Dreyfus et al., 2010). Between 20 and 25 min after wash-in, we could see a stable reduction of the afterdepolarization of single evoked APs by 3 μM TTA-P2, which is therefore partially mediated by T-Type VGCCs (control: *n* = 10/6 cells/animals, TTA-P2: *n* = 5/4 cells/animals, control vs. TTA-P2 at 20–25 min after TTA-P2 wash-in: *p* < 0.01 (*t*-test), Supplementary Figure S1A). Between 20 and 25 min after wash-in, TTA-P2 reduced spine bAP-Ca^2+^ transients significantly compared to time-matched controls (TTA-P2: *n* = 17/6/4 (spines/cells/animals), ctrl: *n* = 24/7/5 (spines/cells/animals), TTA-P2 vs. ctrl: *p* = 0.0002 (*t*-test), Δctrl-TTAP2 = −32.0 ± 23.4%, Figures 1D,K). Experiments were performed at −60 mV (not liquid-junction potential corrected), T-Type channels may be substantially deactivated at this potential. We excluded a major effect of channel deactivation at −60 mV by measuring the effect of TTA-P2 at −75 mV (not liquid-junction potential corrected), which was indistinguishable from the effect at −60 mV (TTA-P2: *n* = 16/6/4 (spines, cells, animals), ctrl: *n* = 21/7/5 (spines, cells, animals), TTA-P2 vs. ctrl: *p* = 0.0011 (*t*-test), Δctrl-TTA-P2 = −22.9 ± 22.5%, Figures 1E,K).

The L-type VGCC antagonist Nimodipine at 20 μM did not reduce spine bAP-Ca^2+^ transients significantly (Nimodipine: *n* = 33/6/3 (spines, cells, animals), ctrl: *n* = 29/7/4 (spines, cells, animals), Nimodipine vs. ctrl: *p* = 0.19, Δctrl-Nimodipine = −13.9 ± 20.0%, Figures 1F,K).

Blocking N-type VGCCs did not have an effect on spine bAP-Ca^2+^ transients, neither with the antagonist PD-173212 at 10 μM (PD-173212: *n* = 15/4/2 (spines, cells, animals), ctrl: *n* = 29/7/4 (spines, cells, animals), PD-173212 vs. ctrl: *p* = 0.92, Δctrl- PD-173212 = −3.7 ± 36.2%, Figures 1G,K) nor with the peptide CtxGIVA at 2 μM (CtxGIVA: *n* = 10/3/2 (spines, cells, animals), ctrl: *n* = 37/8/6 (spines, cells, animals), CtxGIVA vs. ctrl: *p* = 0.20, Δctrl-CtxGIVA = −14.1 ± 12.7%, Figures 1H,K). P/Q VGCC block with AgaIVA (200 nM) also had no significant impact on the bAP-Ca^2+^ transient (AgaIVA: *n* = 19/4/4 (spines, cells, animals), ctrl: *n* = 37/8/6 (spines, cells, animals), AgaIVA vs. ctrl: *p* = 0.09, Δctrl- AgaIVA = −10.8 ± 24.0%, Figures 1I,K). We further hypothesized that store operated Ca^2+^ channels (SOCs) may contribute to the spine bAP-Ca^2+^ transient, given the contribution of Ryanodine Receptor mediated Ca^2+^ release from intracellular stores to bAP Ca^2+^ transients in MEC layer 2 we recently described (Johenning et al., 2015). Using 100 μM of SKF-96365, we did not observe an effect of this unselective SOC blocker (Várnai et al., 2009) on bAP-Ca^2+^ transients (SKF-96365: *n* = 18/5/4 (spines, cells, animals), ctrl: *n* = 29/7/4 (spines, cells, animals), SKF-96365 vs. ctrl: *p* = 0.49, Δctrl- SKF-96365 = −7.5 ± 35.4%, Figures 1J,K). In order to exclude effects of ambient glutamate on NMDARs during bAPs (Wu et al., 2012; but see Herman et al., 2011; Chiu and Jahr, 2017), we also tested the effect of 100 μM D-APV on bAPs. NMDAR block did not affect bAP Ca^2+^ transients significantly in MEC layer 2 neurons (APV: *n* = 15/4/4 (spines, cells, animals), ctrl: *n* = 6/2/2 (spines, cells, animals), APV vs. ctrl: *p* = 0.75, Δctrl- APV = 8.9 ± 34.6%, Supplementary Figure S1B and Figure 1K). We conclude that T- and R-type VGCCs are the two major subtypes on dendritic spines of layer 2 cells in the MEC (Figure 1K). This is in good keeping with a previous study demonstrating that the same VGCC subtypes underly bAP Ca^2+^ transients in dendritic spines of hippocampal CA1 cells (Bloodgood and Sabatini, 2007). The contribution of these two types of VGCCs to bAP-Ca^2+^ transients leads to the question whether and how they could influence synaptic responses.

### Instantaneous Consequences of Specific Block of Spine VGCCs on Synaptic Compound EPSPs

Ligand and voltage-gated channels in the spine head can interact on different timescales. Instantaneous electrical interactions between VGCCs and iGLURs would have an effect on spine excitability and modulate synaptic potentials. At this timescale, VGCCs could contribute to both the depolarization underlying the EPSP and the EPSP Ca^2+^ transient.

Theory suggests that VGCCs could electrically amplify the spine depolarization underlying EPSPs resulting in active local boosting of EPSPs (Koch and Poggio, 1985; Miller et al., 1985; Segev and Rall, 1988). In this context, recruitment of especially low voltage activated T-Type VGCCs by larger synaptic responses has also been discussed (Deisz et al., 1991; Magee et al., 1995; Gillessen and Alzheimer, 1997; Seong et al., 2014). Another acute effect of VGCCs on synaptic potentials was demonstrated in CA1 pyramidal cells, where R-Type Ca^2+^ channels specifically interact with K^+^ channels. Ca^2+^ influx from R-type channels activates K^+^ channels, which results in a dampening of the EPSP, with conflicting results regarding the identity of the target K^+^ channel (Bloodgood and Sabatini, 2007; Giessel and Sabatini, 2011; Wang et al., 2014, 2015). Based on this hypothesis, we used a KMeSO3 intra to enhance the impact of Ca^2+^ activated K^+^ channels, which are reduced by K^+^-gluconate based intracellular solutions (Velumian et al., 1997).

Previously, our experiments demonstrated significantly larger VGCC mediated Ca^2+^ transients in proximal spines of layer 2 cells in the MEC compared to proximal spines in CA1 pyramidal cells (Johenning et al., 2015). Theory predicts that active nonlinear interactions between VGCCs and AMPARs occur in spines with a high VGCC density (Segev and Rall, 1988). Consequently, MEC layer 2 cells are a good model to study the interaction of VGCCs and glutamatergic transmission in spines.

At first, we applied standard extracellular stimulation to test for a contribution of the major VGCC subtypes observed in spines to large synaptically evoked compound EPSPs. With both drugs, there was no significant effect on the first and second EPSP amplitude or the paired pulse ratio (PPR) when compared to time-matched controls (TTA-P2: *n* = 7/5 (cells/animals), ctrl: *n* = 10/6 (cells/animals), EPSP1: *p* = 0.61 (*t*-test), EPSP2: *p* = 0.55 (*t*-test), PPR: *p* = 0.56 (*t*-test), Figure 2A; SNX-482: *n* = 5/3 (cells/animals), ctrl: *n* = 8/6 (cells/animals), EPSP1: *p* = 0.27, EPSP2: *p* = 0.31, PPR: *p* = 0.20, Figure 2B). P/Q-Type Ca^2+^ channel block with AgaIVA and N-Type Ca^2+^ channel block with CtxGIVA resulted in a significant decrease of the evoked compound EPSPs under similar conditions (AgaIVA: *n* = 4/2 (cells/animals), ctrl: *n* = 10/7 (cells/animals), EPSP1: *p* < 0.01 (*t*-test), EPSP2: *p* < 0.01 (*t*-test), Supplementary Figure S2A; CtxGIVA: *n* = 6/4 (cells/animals), ctrl: *n* = 10/7 (cells/animals), EPSP1: *p* < 0.01 (*t*-test), EPSP2: *p* < 0.01 (*t*-test), Supplementary Figure S2B). We conclude that in layer 2 cells of the MEC, T- and R-type VGCCs do not have a direct effect on somatically measured compound synaptic EPSPs.

### Combined Two-Photon Ca^2+^ Imaging and Glutamate Uncaging for Direct Measurements of Single-Spine VGCCs and iGLURs

Previously, we demonstrated that there are large interspine differences with respect to the amplitude of bAP Ca^2+^ transients in layer 2 cells of the MEC. In addition, the responses are much larger when directly compared to proximal spines of hippocampal CA1 cells (Johenning et al., 2015). This variability has not been taken into account by previous studies analyzing the contribution of postsynaptic VGCCs to synaptic transmission and spine electrogenesis, and may be averaged out when stimulating a large population of spines with extracellular stimulation. VGCCs could also affect synaptic plasticity processes on a longer time scale via mechanisms independent of electrical interactions.

We therefore wanted to characterize synaptic properties of single spines with different levels of VGCC mediated Ca^2+^ influx. While it is possible to analyze the Ca^2+^ transients evoked in single spines by extracellular synaptic stimulation, it is not possible to relate these to synaptic properties as extracellular stimulation inevitably recruits several synapses on spines which may not even be in the field of view (Johenning et al., 2009). Two-photon uncaging of glutamate is a tool that permits for selected activation of individual spines (Matsuzaki and Kasai, 2011). We combined two-photon glutamate uncaging, Ca^2+^ imaging and whole-cell patch clamp recordings with a K^+^-gluconate based intracellular solution in which we switched between current clamp and voltage clamp mode. This way, we related the strength of iGLUR mediated uncaging excitatory postsynaptic currents (uEPSCs), interactions between iGLURs and voltage gated conductances during uncaging EPSPs (uEPSPs), uEPSP Ca^2+^ transients and bAP-Ca^2+^ transients at the single spine level (Figure 3A).

A prerequisite for our experiments was a protocol that permitted inter-slice and inter-animal comparisons of spine uEPSC and uEPSP amplitudes in two-photon uncaging experiments. As opposed to slice cultures, where spines are located in a thin layer of tissue, the focal depth of spines in acute slices differs significantly. For inter-experimental comparability, we therefore had to compensate for focal-depth dependent differences in energy deposition of the uncaging laser. The strategy we chose was to use InSpeck microspheres from the same batch, which are standardized fluorescent spheres with a very low inter-sphere variability in fluorophore brightness based on the manufacturer’s specifications (see “Materials and Methods” section). Using identical detection settings between experiments, we positioned the InSpeck microspheres at the tip of a pipette at the same focal plane as our spine of interest (Supplementary Figure S3A). Assuming the InSpeck microsphere’s brightness and the detection efficiency of our two-photon system were constant between different trials, we set the uncaging laser intensity so that we would always detect the same brightness of the bead. This way, we assured identical inter-slice and inter-animal energy deposition of the uncaging laser. We kept inter-experimental caged glutamate concentrations constant by always bath-applying the same concentration of caged glutamate (2.5 mM). For each spine, we located the uncaging spot generating a maximal response. Before each uncaging experiment, the relative position of this uncaging spot and the spine of interest was readjusted to compensate for drift.

Using this approach, we could reproduce the correlation between spine size and uEPSC amplitude demonstrated before in slice cultures (Matsuzaki et al., 2001; Zito et al., 2009) in a population of 50 spines from 30 cells from 21 different animals (*p* = 0.01 and *r* = 0.36, Spearman correlation, Supplementary Figures S3B,D). Using our calibration approach, we generated uEPSCs with an amplitude of 26.03 ± 1.54 pA (mean ± SEM). This is in the same range as the unitary amplitudes of local inputs onto layer 2 cells in the MEC we measured previously in age-matched rats (Beed et al., 2010). These local inputs correspond to the proximal apical spines under investigation here (Ma et al., 2008). In the same spine population, we correlated neck length with the EPSC amplitude but could not find a significant correlation (*p* = 0.07 and *r* = −0.25, Spearman correlation, Supplementary Figures S3B,C).

### NMDAR Contribution to uEPSPs Evoked by Two-Photon Uncaging

In order to measure the impact of VGCCs on spine Ca^2+^ transients evoked by glutamate uncaging, we switched to the current clamp mode. In current clamp, local depolarization upon synaptic stimulation relieves the Mg^2+^ block of NMDARs (Nevian and Sakmann, 2004). The result is a higher activation of spine NMDARs in current clamp than in voltage clamp. Given the higher glutamate affinity of NMDARs in comparison to AMPARs (Pankratov and Krishtal, 2003), we first wanted to test for the NMDAR contribution to our uncaging EPSP (uEPSP) signals. When measuring in voltage clamp, the uEPSCs measured in the spine population under control conditions were indistinguishable from the spines preincubated in the NMDAR-blocker APV (APV: *n* = 8/4/4 (spines/cells/animals), 19.8 ± 5 pA, Control: *n* = 16/14/10 (spines/cells/animals), 19.2 ± 7.5 pA, *p* = 0.96 (*t*-test), Supplementary Figure S4B). When switching to current clamp conditions in the same population of spines, uEPSPs were significantly smaller in the APV preincubated group (APV: *n* = 8/4/4 (spines/cells/animals), 0.54 ± 0.21 mV, Control:*n* = 16/14/10 (spines/cells/animals), 0.80 ± 0.43 mV, *p* = 0.045 (*t*-test), Supplementary Figure S4A). This was also reflected by a significant reduction of the uEPSP mediated Ca^2+^ transients in APV measured with 500 μM Fluo-5F (APV: *n* = 17/8/4 (spines/cells/animals), 0.044 ± 0.027 G/G_max_, Control: *n* = 12/11/8 (spines/cells/animals), 0.115 ± 0.074 G/G_max_, *p* = 0.0002, Supplementary Figure S4C), which we could not observe when comparing bAP-Ca^2+^ transients (APV: *n* = 22/8/4 (spines/cells/animals), 0.113 ± 0.063 G/G_max_, Control: *n* = 26/16/10 (spines/cells/animals), 0.131 ± 0.087 G/G_max_, *p* = 0.50, Supplementary Figure S4D).

We next wanted to find out whether we could observe a comparable APV effect on the synaptically evoked compound EPSP. Here, we again compared APV-wash-in to time-matched controls. However, as opposed to our uncaging experiments, APV did not have a significant effect on the synaptically evoked first or second compound EPSP amplitude (APV: *n* = 8/4 (cells/animals), ctrl: 10/6 (cells/animals), EPSP1: *p* = 0.18 (*t-test*), EPSP2: *p* = 0.24 (*t*-test), Supplementary Figure S4E). This means that all NMDAR-related measures described here when using two-photon uncaging do not reflect synaptic NMDARs exclusively but also depend on the extrasynaptic NMDARs on a spine. This has to be kept in mind when comparing uEPSPs and uEPSCs to synaptic activation by extracellular stimulation. The physiological relevance of this extrasynaptic pool of spine NMDARs is not clear. Extrasynaptic NMDARs on spines have been proposed to get recruited by synaptic glutamate spillover during repetitive synaptic inputs (Mainen et al., 1999).

### Instantaneous Effect of VGCCs on Local Ca^2+^ Transients in Dendritic Spines

To test whether VGCC expression in spines is linked to their synaptic properties, we obtained a dataset with single spine measurements of bAP-Ca^2+^ transients, EPSP related Ca^2+^ transients, somatic EPSP amplitudes and somatic EPSC amplitudes measured in voltage clamp (Figure 3A). We measured spine Ca^2+^ transients with the low affinity Ca^2+^ indicator Fluo-4FF. In contrast to the pharmacology experiments in Figure 1, we used a low-affinity Ca^2+^ indicator in order to minimize dye nonlinearities that could occur when measuring synaptic Ca^2+^ transients (Yasuda et al., 2004).

Previous work shows that VGCCs locally enhance the EPSP related Ca^2+^ influx on the single spine level. This local influx was not mirrored by VGCC related changes of the somatic EPSP (Bloodgood et al., 2009; Seong et al., 2014; Bywalez et al., 2015).

Probing with bAPs, we can distinguish between spines with different levels of VGCC mediated Ca^2+^ influx. The previously demonstrated absence of a correlation between bAP-Ca^2+^ transient amplitude and spine morphology leaves VGCC activation levels as the most likely explanation for spine to spine differences in bAP-Ca^2+^ transients (Johenning et al., 2015).

First, we tested whether the synaptic Ca^2+^ transient depends on the amount of synaptically mediated depolarization. When plotting the uEPSP amplitude against the uEPSP Ca^2+^ transient, we did not see a correlation (*n* = 22/11/9 (spines/cells/animals), *r* = 0.102, *p* = 0.65, Figure 3B). To find out what determines the size of the uEPSP Ca^2+^ transient, we first wanted to test if the VGCC content assessed by bAP-Ca^2+^ transients can explain differences in local glutamate mediated spine Ca^2+^ influx. However, these two values were not correlated (*n* = 22/10/9 (spines/cells/animals), *p* = 0.92, *r* = −0.023, Spearman correlation, Figure 3C). Nevertheless, in APV we still observe Ca^2+^ influx upon glutamate uncaging (Supplementary Figure S4C). From this we conclude that while VGCCs can contribute to uEPSP-related Ca^2+^ influx, they do not determine its amplitude.

Theoretical studies predict that large local synaptic depolarizations which would significantly recruit additional voltage gated conductances are preferentially reached in spines with a large spine neck resistance (Segev and Rall, 1988). To test this hypothesis, we related the uEPSP Ca^2+^ transient as a proxy for local depolarization to the spine neck length. As can be seen in Figure 3D, spine neck length does not correlate with the uEPSP Ca^2+^ transient (*n* = 22/11/9 (spines/cells/animals), *p* = 0.49, *r* = 0.15, Spearman correlation, Figure 3D). Our experiments therefore do not permit the conclusion that there may be a strong VGCC mediated contribution to the uEPSP spine Ca^2+^ transient in spines with a larger neck resistance. In sum, our data permits the conclusion that on the single spine level, spine VGCCs do not actively contribute to spine electrogenesis in the form of spine-specific Ca^2+^ spikes.

### Long-Term Interactions Between VGCCs and Ionotropic GLURs

bAPs may still contribute to AMPAR mediated transmission beyond the level of direct electrical interactions. This interaction would occur on the level of plasticity processes. Synaptic plasticity changes the synaptic weight on a longer time scale that outlasts the timeframe of individual stimuli (Korte and Schmitz, 2016). The amplitude of the bAP-Ca^2+^ transient is inversely related to the induction of LTD induced by NMDAR activation (Hayama et al., 2013). In addition, bAP-burst mediated Ca^2+^ transients and NMDARs interact during the induction of LTP (Kampa, 2006). A recent study has proposed a central role for bAP-mediated VGCC activation for the induction of synaptic plasticity (Tigaret et al., 2016).

We hypothesized that a spine’s VGCC content reflected by the amplitude of bAP-Ca^2+^ transients should correlate with synaptic strength if these parameters interact directly and significantly. In order to test whether there is a relationship between the AMPAR response in voltage clamp and the bAP-Ca^2+^ transient, we measured the bAP-Ca^2+^ transient and the uEPSC in individual spines (Figure 3E). However, there is no significant correlation between the two parameters (*n* = 33/15/11 (spines/cells/animals), *p* = 0.95, *r* = 0.01, Spearman correlation, Figure 3E). So far, we used synaptic stimulation (Figure 2) and two-photon uncaging (Figure 3) to probe for AMPARs at the single spine level. Based on these sets of experiments, we conclude that fast AMPAR mediated synaptic transmission and VGCCs are functionally uncoupled.

In addition to synaptic AMPARs, NMDARs can also be bidirectionally modified by patterned synaptic stimulation (Rebola et al., 2010; Hunt et al., 2013). NMDARs and VGCCs are the main Ca^2+^ sources during neuronal activity and together determine the amount of Ca^2+^ entering the spine during synaptic activity. We therefore wanted to test whether the number of VGCCs on a spine could relate to a single spine’s NMDAR pool.

As a measure of a spine’s NMDAR pool, we switched to voltage clamp measurements using a Caesium-based intracellular solution and TTX to optimize voltage control. We measured AMPAR based uEPSCs (uAMPA) at −60 mV (Figure 4A2) and NMDAR driven uEPSCs (uNMDA) at +40 mV (Figure 4A3). The amplitude of the NMDAR uEPSC was measured 50–60 ms after stimulus onset to avoid contamination by AMPA currents. In order to accentuate the contribution of synaptic NMDARs to our uncaging evoked currents, the synaptic NMDAR-specific coagonist Serine was added in these experiments (Papouin et al., 2012). Under these conditions optimized for voltage clamp recordings, bAPs cannot be evoked by current injection. To probe for single-spine VGCCs, we applied 30 ms voltage steps from −60 mV to 0 mV to measure the VGCC mediated Ca^2+^ influx (Figure 4A4). Similar to the results when using a K^+^-based intracellular solution, the AMPAR-mediated uEPSC was not related to the single-spine VGCC mediated Ca^2+^ influx (*n* = 16/9/6 (spines/cells/animals), *p* = 0.09, *r* = −0.44, Spearman correlation, Figure 4B). When grouping the spines by the median of the VGCC mediated Ca^2+^ influx, there was also no significant difference in AMPA-current between the small VGCC Ca^2+^response spines and the large VGCC Ca^2+^response spines (*p* = 0.51 (*t*-test)). However, there was a significant inverse correlation between the NMDAR mediated current at +40 mV and the VGCC mediated Ca^2+^ influx (*n* = 16/9/6, *p* = 0.04, *r* = −0.52, Spearman correlation, Figure 4C). When grouping the spines by the median of the depolarization induced Ca^2+^ transient, there was a significantly smaller NMDAR current in the group with the larger VGCC mediated Ca^2+^ influx (*p* = 0.013; *t-test*).

### Effect of Spine VGCC Enhancement on NMDARs

Spines with large bAP-Ca^2+^ transients could therefore undergo a reduction in NMDARs. In our previous work, we demonstrated that suprathreshold activity results in long-lasting enhancement of bAP-Ca^2+^ transients. We next wanted to test if bAP-Ca^2+^ transients can be directly related to a reduction in NMDARs. In this experiment, we wanted to test if NMDAR-mediated currents evoked by extracellular synaptic stimulation can be modulated by period of bAP bursts previously demonstrated to enhance spine VGCCs (Johenning et al., 2015). A K^+^-gluconate based intracellular solution was used so that bAPs could be evoked. To relieve the Mg^2+^ block of the NMDAR at hyperpolarized membrane potentials we used a low Mg^2+^extracellular solution. AMPARs were blocked with NBQX. We then stimulated synaptically using an extracellular stimulation electrode. Single presynaptic pulses do not saturate the postsynaptic spine NMDARs (Svoboda and Mainen, 1999). This lack of saturation could be attributed to the recruitment of extrasynaptic NMDARs by repetitive stimuli (Pankratov and Krishtal, 2003; Harris and Pettit, 2008). For compatibility with our two-photon uncaging experiments, where we most likely also stimulated extrasynaptic NMDARs, we stimulated with double pulses and focussed our analysis on the second EPSC in a double pulse (Figure 5A). We then compared the effect of a 5 min burst of bAPs to a control group that did not fire APs. The effect of the bAPs and the control interval are normalized to the pre-induction baseline.

As predicted from the correlative data in Figure 4, in comparison to no-AP controls, AP firing resulted in significantly smaller NMDAR-currents when compared to controls at 30–35 min after the induction interval (5AP train: EPSP2 = 128.6 ± 54.9%, *n* = 8/7 (cells/animals), control: EPSP2 = 97.5 ± 20.1%, *n* = 10/9 (cells/animals), EPSP2 vs. ctrl: *p* = 0.018 (Wilcoxon-Mann-Whitney *U* Test, one-tailed, Figures 5B,C).

## Discussion

Ca^2+^ is a major player in long-term synaptic adaptations underlying plasticity. Depolarization of dendritic spines activates VGCCs, both by direct synaptic activation and by electrotonic spread of depolarization mediated by dendritic bAPs. Here, we demonstrate that the major VGCC subtypes contributing to bAP Ca^2+^ transients in layer 2 cells of the MEC are R- and T-type channels. R- and T-type channel activation by synaptic activation of single spines contributes to the spine Ca^2+^ response, but does not contribute to the depolarization underlying EPSPs.

In addition to direct interactions on a shorter time scale, VGCCs may also affect the strength of ionotropic glutamatergic signaling on a longer time scale by interfering with plasticity processes. While there is no relationship between spine VGCCs and AMPARs, VGCC-mediated transients are inversely correlated with the NMDAR current of individual spines. In addition, bAP-mediated Ca^2+^ transients induce downscaling of NMDAR currents. These two findings demonstrate a dose-dependent global modification of the spine NMDAR content by VGCC mediated Ca^2+^ influx on a longer timescale. Our study provides a direct link between global VGCC activation by dendritic backpropagation of APs and synaptic function. The activity dependent selective downscaling of NMDARs could serve to homeostatically stabilize glutamatergic synapses on active cells by increasing their plasticity threshold without scaling down AMPAR-mediated synaptic strength.

### Effects of Depolarization Mediated VGCC Ca^2+^ Influx on Synaptic Potentials

Previous studies have generated mixed results regarding the direct contribution of VGCCs to synaptic depolarization. Some reports using extracellular synaptic stimulation suggest that there is direct boosting of large compound EPSPs by VGCCs (Magee and Johnston, 1995; Gillessen and Alzheimer, 1997). Voltage imaging of EPSPs in dendritic spines suggests that there is no significant VGCC contribution to local synaptic depolarization (Palmer and Stuart, 2009; Popovic et al., 2015). Here, we utilized drugs that are highly selective for the two major postsynaptic VGCC subtypes in dendritic spines of layer 2 cells in the MEC (Figure 1). We could therefore directly test VGCC contribution to large compound EPSPs evoked by direct extracellular stimulation. This approach also takes cooperative effects between several synapses into account. Our results show that in MEC layer 2 cells, the amount of depolarizing currents added to the compound synaptic potential by spine VGCCs is too small to play a quantitatively significant role.

The absence of a T- and R-type VGCC block mediated effect on synaptic depolarization measured at the soma is also relevant with respect to another interaction between VGCCs and EPSPs: in CA1 pyramidal cells, R-type VGCCs initiate a negative feedback loop by activating Ca^2+^ activated K^+^ channels. This decreases the EPSP amplitude in single spines (Bloodgood and Sabatini, 2007; Giessel and Sabatini, 2011; Wang et al., 2014, 2015). In layer 5 prefrontal cortex neurons, compound EPSPs are dampened by this negative feedback loop, whereas single spine uEPSPs are not affected (Seong et al., 2014). In excitatory layer 2 neurons of the MEC, the VGCC-mediated negative feedback loop described in other principal neurons is absent even in compound EPSPs. It seems that the degree of coupling between VGCCs and Ca^2+^ activated K^+^ channels with an effect on the EPSP is cell-type specific and does not occur in all excitatory neurons in the forebrain.

So far, we focussed our discussion on instantaneous interactions between VGCCs and iGLURs when measuring EPSPs at the cell soma. However, this might have filtered out a VGCC-mediated boosting of the EPSP in spines (Harnett et al., 2012; Acker et al., 2016). Therefore, the limited electrogenic role of VGCCs when measuring synaptic activity at the soma does not rule out the local contribution of VGCCs to synaptic responses. This way, VGCCs could still shape and determine local EPSP Ca^2+^ transients.

Our results using two-photon uncaging for glutamatergic stimulation are not compatible with local non-linear electrogenic effects of VGCCs in single spines of layer 2 MEC neurons. Our experimental results in APV clearly suggest that VGCCs contribute to EPSP Ca^2+^ transients (Supplementary Figure S4C). This contribution needs to be considered from a quantitative perspective. We therefore tested whether the quantitative variability observed in spine VGCC content could explain the variability of the uncaging-evoked spine Ca^2+^ response. From our experiments, we conclude that the amplitude of EPSP Ca^2+^transients is regulated independent of VGCC expression. This hints at a dominant role for the interaction of AMPARs and NMDARs in setting the magnitude of the EPSP Ca^2+^transient. These findings further argue against a significant local effect of VGCCs in synaptic depolarization and against local active processes like single-spine Ca^2+^ spikes.

### Long-Term Effects of Depolarization Mediated VGCC Ca^2+^ Influx by bAPs

Alternatively, while contributing to EPSP Ca^2+^ transients, the main functional relevance of VGCCs may only be mediated by bAP-Ca^2+^ transients. This interaction would occur on a more prolonged timescale. Exclusive VGCC activation by electrotonic spread could permit for a distinction between local specific mechanisms in synaptically driven spines and global unspecific mechanisms in spines that are depolarized but not synaptically driven.

In synaptically driven spines, a recent study has identified a central role for bAP-mediated VGCC activation for the induction of synaptic plasticity (Tigaret et al., 2016). In this study, partial block of VGCCs with different pharmacological compounds could not block the induction of synaptic plasticity, only a cocktail of VGCC blockers incorporating R-, T- and L-type VGCCs had an effect. Our interpretation is that while coactivation of VGCCs *per se* is a prerequisite for plasticity induction, the range of VGCC-mediated [Ca^2+^] for plasticity induction is rather large. This large safety margin for VGCC recruitment during plasticity induction is reflected by our finding that AMPAR mediated synaptic currents are independent of the physiological range of the VGCC mediated bAP-Ca^2+^ transient and the depolarization mediated spine Ca^2+^ influx measured in voltage clamp (Figures 3, 4).

What happens to synapses that are not synaptically activated but experience bAP-Ca^2+^ transients when a neuron receives suprathreshold activation? Our results suggest that spine AMPARs and, by extension, AMPAR plasticity are independent of a spine’s VGCC content and the related bAP-Ca^2+^ transient. In addition to AMPARs, NMDARs are involved in the induction of both synaptic depression and potentiation (Malenka and Bear, 2004). In CA3 neurons, bidirectional hebbian plasticity of NMDARs has been demonstrated to increase or decrease the threshold for LTP induction (Rebola et al., 2011; Hunt et al., 2013). In the context of homeostatic plasticity, NMDARs are globally upregulated by long phases of reduced network activity and globally downregulated by long phases of enhanced network activity in neuronal cultures (Watt et al., 2000). Here, we contribute a new mechanism of global activity-dependent NMDAR regulation: the activity-dependent enhancement of bAP-Ca^2+^ transients by bAP mediated depolarization results in the downregulation of NMDAR function. We infer this from two observations: first, on the single spine level the NMDAR content is inversely correlated with the VGCC mediated depolarization associated Ca^2+^ influx (Figure 4). Accordingly, the degree of NMDAR downscaling in a spine seems to depend on the amplitude of the depolarization associated Ca^2+^ influx. Second, AP bursts result in downscaling of NMDARs when compared to controls that do not experience AP firing during the induction period (Figure 5). Based on methodological constraints of two-photon uncaging, we cannot differentiate synaptic NMDARs from spine NMDARs located extrasynaptically (Supplementary Figure S4) when measuring uEPSPs and uEPSCs. We added 10 μM of the NMDAR-coagonist Serine in our voltage-clamp experiments when we quantified the relationship between step depolarization mediated Ca^2+^ influx and NMDAR currents (Figure 4). It has previously been published that Serine predominantly activates synaptic NMDARs (Papouin et al., 2012). In addition, experiments in Figure 5 demonstrate that synaptic NMDAR-EPSCs evoked by synaptic stimulation are functionally reduced by AP firing. This implies a causal link between AP firing, dendritic backpropagation and downscaling of synaptic and extrasynaptic NMDARs on spines. So far and to the best of our knowledge, the bAP-Ca^2+^ transient is the only biochemical transducer of bAP mediated spine depolarization. The most parsimonious explanation therefore is that bAP-Ca^2+^ transients induce downregulation of spine NMDARs in a dose-dependent manner.

In this context, we would like to compare LTP induction, synaptic NMDAR content and bAP mediated Ca^2+^ influx between layer 2 cells in the MEC and hippocampal CA1 neurons. Layer 2 cells in the MEC have larger bAP-Ca^2+^ transients than CA1 pyramids (Johenning et al., 2015). Interestingly, this is accompanied by a smaller NMDAR/AMPAR ratio and difficulties in LTP induction in MEC layer 2 cells of rats in the same age range (Deng and Lei, 2007).

Future experiments will have to show whether the downregulation of NMDARs indeed has a metaplastic effect on synaptic plasticity by shifting the induction threshold and occurs homeostatically in response to reduced or elevated activity levels of individual neurons in a network. In this case, downregulation of NMDARs by bAPs may serve as a homeostatic mechanism specific for synapses that have not been involved in the suprathreshold activation of individual neurons. This heterosynaptic adaptation would result in potentiation of only a subset of repeatedly activated synapses, while less frequently activated synapses would have a higher plasticity threshold. This type of metaplasticity would mainly affect dendritic spines that have not been specifically activated. We therefore propose a stabilizing mechanism that does not interfere with information transfer. In this model, silent neurons would keep high levels of spine NMDARs, constituting a pool of plasticity competent neurons that can be integrated in the network by novel activation patterns.

## Author Contributions

AKT and FWJ performed experiments and analyzed the data. AKT, FWJ, DS, GK and BR conceptualized the research, designed experiments, interpreted the data, wrote the manuscript and prepared the figures. GK and BR contributed reagents.

## Conflict of Interest Statement

GK and BR are founders of Femtonics Kft and BR is a member of its scientific advisory board. The other authors declare that the research was conducted in the absence of any commercial or financial relationships that could be construed as a potential conflict of interest.

## Footnotes

^1^https://www.graphpad.com/scientific-software/prism/

## Supplementary Material

FIGURE S1 **(A1)** Averaged afterdepolarization (ADP) traces 0–5 min and after wash-in of 3 μM TTA-P2 (25–30 min) of a control experiment (black) and when TTA-P2 is washed-in after a 5 min baseline (green). **(A2)** Time plot of the normalized, binned (1 min) afterdepolarization of an evoked AP under control conditions (black) and when 3 μM TTA-P2 (green) is washed-in after 5 min of baseline. **(B)** Time plots of normalized, binned (3 min) doublet evoked bAP-Ca^2+^ transients under control conditions (black) and wash-in of 100 μM APV (light red).

FIGURE S2 Reduction of synaptically evoked compound EPSPs measured at the soma by N- and P/Q-type VGCC block.** (A1,B1)** Extracellular synaptic paired-pulse stimulation evoked compound EPSPs under baseline conditions (black) and after wash-in of 200 nM AgaIVA (purple) and 1 μM CtxGIVA (blue) 20–25 min after wash-in. **(A2,A3,B2,B3)** Time plots of normalized, binned (1 min) maximum amplitudes of EPSP1 **(A2,B2)** and EPSP2** (A3,B3)** under control conditions (black) and during wash-in of 200 nM AgaIVA (purple) and 1 μM CtxGIVA (blue).

FIGURE S3 Calibration of uncaging laser intensity to compensate for interslice variability. **(A1)** Image of the target dendritic segment (white arrow) and the InSpeck microsphere in close proximity (white square). Scale bar: 50 μm. **(A2)** Intensity measurement of the bead (light intensity measured in A.U.). Inset: magnification of the bead and illustration of the line for determination of the intensity (yellow line). **(B1)** Z-projection of an imaged dendritic segment. Scale bar: 2 μm. **(B2)** uEPSCs corresponding to the spines marked in **(B1)**. **(C)** Correlation of neck length and uEPSC. **(D)** Correlation of spine size and uEPSC. Statistics: spearman correlation. **(D)** Correlation of spine size and uEPSC. Statistics: spearman correlation.

FIGURE S4 Activation of NMDARs by two-photon uncaging but not by electrical synaptic stimulation.** (A1)** Uncaging evoked EPSPs from different spines under control conditions (left panel, mean is depicted in black) and preincubated in D-APV (right panel, mean is depicted in black). **(A2)** Median boxplot of uncaging evoked uEPSPs under control conditions and in D-APV. Statistics: Wilcoxon-Mann-Whitney Test. **(B1)** Uncaging evoked EPSCs under control conditions (left panel, mean is depicted in black) and in D-APV (right panel, mean is depicted in black). **(B2)** Median boxplot of uncaging evoked uEPSCs under control conditions and in D-APV. Statistics: Wilcoxon-Mann-Whitney Test. **(C1)** Uncaging evoked Ca^2+^ transients under control conditions (left panel, mean is depicted in black) and in D-APV (right panel, mean is depicted in black) from the same spines as in **(A)**. **(C2)** Median boxplot of the uncaging evoked Ca^2+^ transients depicted in **(C1)**. Statistics: Wilcoxon-Mann-Whitney Test. **(D1)** bAP-doublet evoked Ca^2+^ transients under control conditions (left panel, mean is depicted in black) and in D-APV (right panel, mean is depicted in black). **(D2)** Median boxplot of bAP-doublet evoked Ca^2+^ transients under control conditions and in D-APV. Statistics: Wilcoxon-Mann-Whitney Test. **(E1)** Paired-pulse extracellular synaptic stimulation evoked compound EPSPs under control conditions (black) and 20–25 min after wash-in of 100 μM D-APV (red). **(E2,E3)** Time plots of normalized, binned (1 min) maximum amplitudes of EPSP1 **(E2)** and EPSP2 **(E3)** under control conditions (black) and during wash-in of D-APV (red).

FIGURE S5 Pie charts of age distributions. The pie charts illustrate the age distribution of the animals measured in our experiments ordered by the figures in which they appear.
