# Supplementary material for: Voltage Gated Calcium Channel Activation by Backpropagating Action Potentials Downregulates NMDAR Function
Source: Front Cell Neurosci. 2018 Apr 23;12:109. doi: 10.3389/fncel.2018.00109 (PMC5932410; doi:10.3389/fncel.2018.00109)
Supplement: Supplementary file 1 [file Table_1.DOCX]

**per cell**

Supplementary table Fig. 1

|  | **n-number**  (per cell) | **Resting membrane potential**  (mean ± SEM) | **Holding current**  (mean ± SEM) |
| --- | --- | --- | --- |
| Ni^2+^ (1mM) | 5 | -57.4 ± 1.0 | -29.0 ± 24.4 |
| Ni^2+^ (100µM) | **6** | -58.4 ± 1.0 | -0.1 ± 22.9 |
| Ctrl (Ni^2+^) | 5 | -58.7 ± 1.0 | -1.3 ± 24.2 |
| SNX-482 | 5 | -55.5 ± 0.7 | -55.1 ± 11.0 |
| TTA-P2 (-60mV) | 6 | -57.9 ± 0.9 | -30.8 ± 22.1 |
| Ctrl TTA-P2 (-60mV) | 7 | -56.7 ± 0.8 | -55.6 ± 20.1 |
| TTA-P2 (-75mV) | 6 | -57.9 ± 0.9 | -30.8 ± 22.1 |
| Ctrl TTA-P2 (-75mV) | 7 | -56.7 ± 0.8 | -55.6 ± 20.1 |
| Nimodipine | 6 | -58.1 ± 1.3 | -8.2 ± 19.9 |
| PD-173212 | 4 | -56.1 ± 0.9 | -8.2 ± 19.9 |
| CtxGIVA | 3 | -59.2 ± 2.9 | 4.7 ± 57.3 |
| AgaIVA | 4 | -58.5 ± 0.6 | 5.7± 15.0 |
| SKF-96365 | 5 | -57.3 ± 1.1 | -22.2 ± 24.7 |
| Ctrl (SNX-482, CtxGIVA and AgaIVA) | 8 | -57.4 ± 0.7 | -30.0 ± 15.9 |
| Ctrl (Nimodipine, PD-173212 and SKF-96365) | 7 | -56.5 ± 0.7 | -49.9 ± 19.5 |

| All experiments (cells)  *Cells were several parameters are recorded from are only counted once | 71 | -57.45 ± 0.3 | -26.7 ± 6.1 |
| --- | --- | --- | --- |

Supplementary table Fig. S3 a-d

|  | **n-number**  (per cell) | **Resting membrane potential**  (mean ± SEM) | **Holding current**  (mean ± SEM) |
| --- | --- | --- | --- |
| uEPSP (Ctrl) | 14 | -59.4 ± 0.5 | 24.7 ± 13.4 |
| uEPSP (APV) | 4 | -58.1 ± 1.7 | 15.9 ± 25.4 |
| uEPSC (Ctrl) | 14 | -59.4 ± 0.5 | 24.7 ± 13.4 |
| uEPSC (APV) | 4 | -58.1 ± 1.7 | 15.9 ± 25.4 |
| ΔG/G_max_ uEPSP (Ctrl) | 11 | -59.6 ± 0.6 | 21.1 ± 14.8 |
| ΔG/G_max_ uEPSP (APV) | 8 | -58.3 ± 0.9 | 20.2 ± 16.1 |
| ΔG/G_max_ 2bAPs (Ctrl) | 16 | -59.6 ± 0.5 | 27.0 ± 11.8 |
| ΔG/G_max_ 2bAPs (APV) | 8 | -58.3 ± 0.9 | 20.2 ± 16.1 |

| All experiments (cells)  *Cells were several parameters are recorded from are only counted once | 23 | -59.1 ± 0.5 | -23.9 ± 9.7 |
| --- | --- | --- | --- |

Supplementary table Fig. S3 e

|  | **n-number**  (per cell) | **Resting membrane potential**  (mean ± SEM) | **Holding current**  (mean ± SEM) |
| --- | --- | --- | --- |
| EPSP (Ctrl) | 10 | -61.2 ± 0.9 | -46.9 ± -29.1 |
| EPSP (APV) | 8 | -62.2 ± 0.5 | -29.1 ± 22.0 |

| All experiments (cells) | 18 | -61.7 ± 0.5 | -39.0 ± 14.3 |
| --- | --- | --- | --- |

**per spine**

Supplementary table Fig. 1

|  | **n-number**  (per cell) | **Resting membrane potential**  (mean ± SEM) | **Holding current**  (mean ± SEM) |
| --- | --- | --- | --- |
| Ni^2+^ (1mM) | 19 | -57.0 ± 0.5 | -39.2 ± 11.5 |
| Ni^2+^ (100µM) | 21 | -58.0 ± 0.5 | -3.6 ± 12.7 |
| Ctrl (Ni^2+^) | 22 | -58.8 ± 0.4 | -1.4 ± 10.6 |
| SNX-482 | 16 | -55.5 ± 0.4 | -61.4 ± 6.9 |
| TTA-P2 (-60mV) | 16 | -58.0 ± 0.5 | -37.1 ± 12.6 |
| Ctrl TTA-P2 (-60mV) | 24 | -56.3 ± 0.4 | -51.4 ± 8.2 |
| TTA-P2 (-75mV) | 16 | -57.9 ± 0.6 | -33.7 ± 12.9 |
| Ctrl TTA-P2 (-75mV) | 21 | -56.2 ± 0.4 | -71.8 ± 10.4 |
| Nimodipine | 33 | -57.8 ± 0.6 | -11.3 ± 8.6 |
| PD-173212 | 15 | -56.2 ± 0.4 | -55.7 ± 12.1 |
| CtxGIVA | 10 | -59.8 ± 1.4 | 16.1 ± 28.1 |
| AgaIVA | 19 | -58.5 ± 0.3 | 4.2± 6.5 |
| SKF-96365 | 18 | -57.5 ± 0.5 | -17.9 ± 11.5 |
| Ctrl (SNX-482, CtxGIVA and AgaIVA) | 37 | -57.6 ± 0.3 | -25.1 ± 6.3 |
| Ctrl (Nimodipine, PD-173212 and SKF-96365) | 29 | -56.3 ± 0.3 | -58.1 ± 8.9 |

| All experiments (spines)  *Spines were several parameters are recorded from are only counted once | 280 | -57.5 ± 0.1 | -26.4 ± 3.1 |
| --- | --- | --- | --- |

Supplementary table Fig. S3 a-d

|  | **n-number**  (per spine) | **Resting membrane potential**  (mean ± SEM) | **Holding current**  (mean ± SEM) |
| --- | --- | --- | --- |
| uEPSP (Ctrl) | 16 | -59.3 ± 0.5 | 25.8 ± 12.6 |
| uEPSP (APV) | 8 | -58.8 ± 0.8 | 25.6 ± 13.1 |
| uEPSC (Ctrl) | 16 | -59.3 ± 0.5 | 25.8 ± 12.6 |
| uEPSC (APV) | 8 | -58.8 ± 0.8 | 25.6 ± 13.1 |
| ΔG/G_max_ uEPSP (Ctrl) | 12 | -59.7 ± 0.5 | 26.4 ± 14.5 |
| ΔG/G_max_ uEPSP (APV) | 17 | -58.5 ± 0.6 | 21.9 ± 10.4 |
| ΔG/G_max_ 2bAPs (Ctrl) | 26 | -59.7 ± 0.4 | 26.3 ± 8.9 |
| ΔG/G_max_ 2bAPs (APV) | 22 | -58.9 ± 0.5 | 29.5 ± 8.7 |

| All experiments (spines)  *Spines were several parameters are recorded from are only counted once | 45 | -59.3 ± 0.3 | 28.3 ± 6.5 |
| --- | --- | --- | --- |
