# Supplementary figures and images for: Voltage Gated Calcium Channel Activation by Backpropagating Action Potentials Downregulates NMDAR Function
Source: Front Cell Neurosci. 2018 Apr 23;12:109. doi: 10.3389/fncel.2018.00109 (PMC5932410; doi:10.3389/fncel.2018.00109)

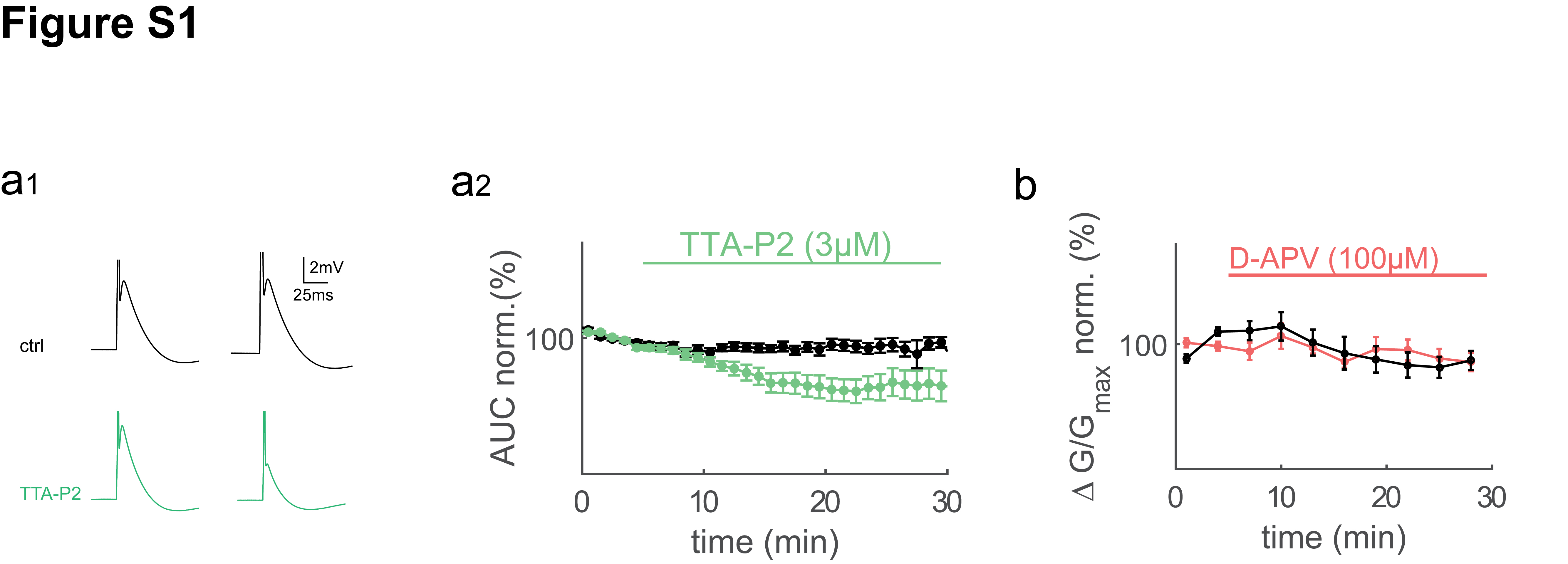

Supplement: FIGURE S1 — (A1) Averaged afterdepolarization (ADP) traces 0–5 min and after wash-in of 3 μM TTA-P2 (25–30 min) of a control experiment (black) and when TTA-P2 is washed-in after a 5 min baseline (green). (A2) Time plot of the normalized, binned (1 min) afterdepolarization of an evoked AP under control conditions (black) and when 3 μM TTA-P2 (green) is washed-in after 5 min of baseline. (B) Time plots of normalized, binned (3 min) doublet evoked bAP-Ca2+ transients under control conditions (black) and wash-in of 100 μM APV (light red). [file Image_1.JPEG]

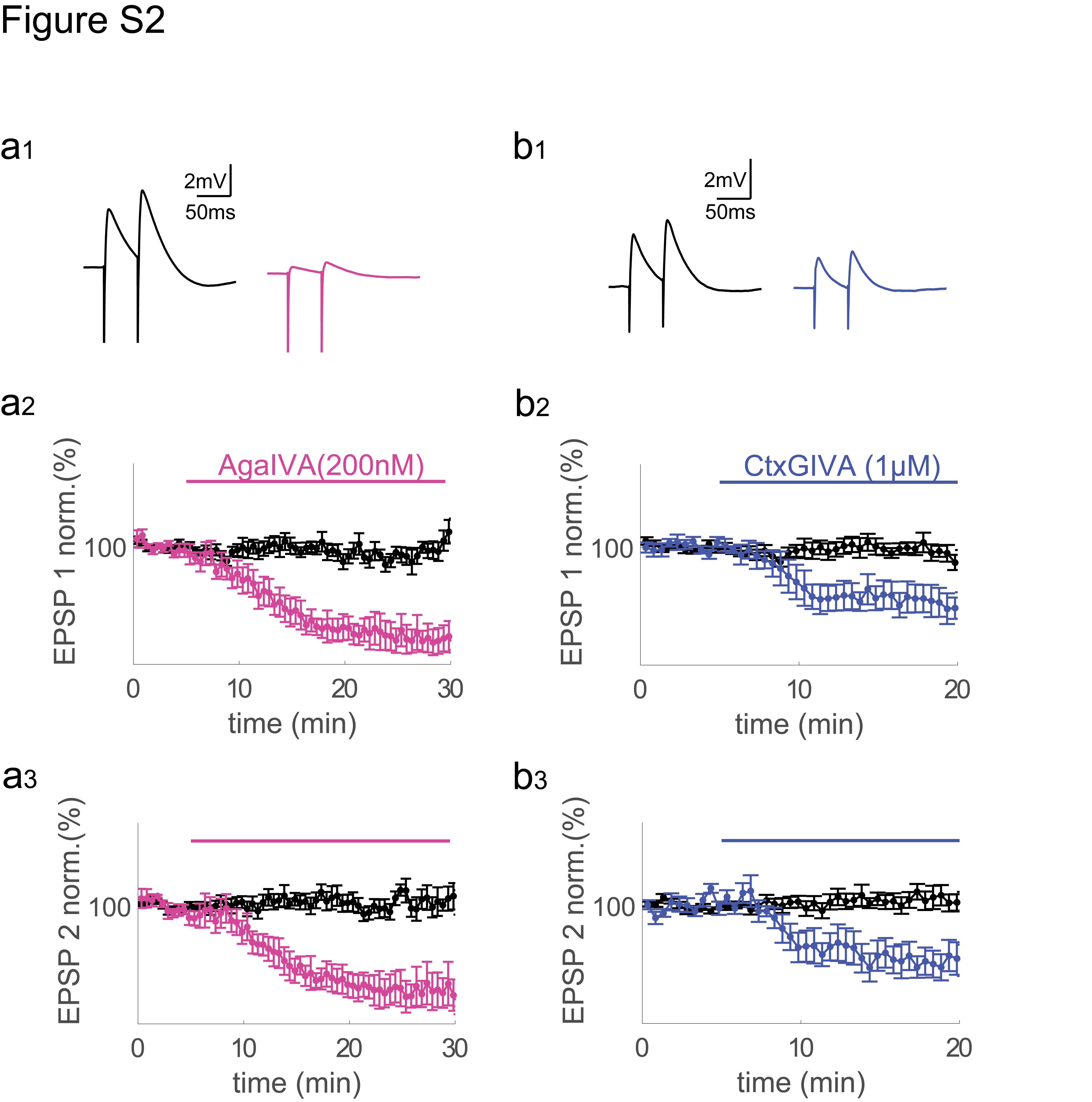

Supplement: FIGURE S2 — Reduction of synaptically evoked compound EPSPs measured at the soma by N- and P/Q-type VGCC block. (A1,B1) Extracellular synaptic paired-pulse stimulation evoked compound EPSPs under baseline conditions (black) and after wash-in of 200 nM AgaIVA (purple) and 1 μM CtxGIVA (blue) 20–25 min after wash-in. (A2,A3,B2,B3) Time plots of normalized, binned (1 min) maximum amplitudes of EPSP1 (A2,B2) and EPSP2 (A3,B3) under control conditions (black) and during wash-in of 200 nM AgaIVA (purple) and 1 μM CtxGIVA (blue). [file Image_2.JPEG]

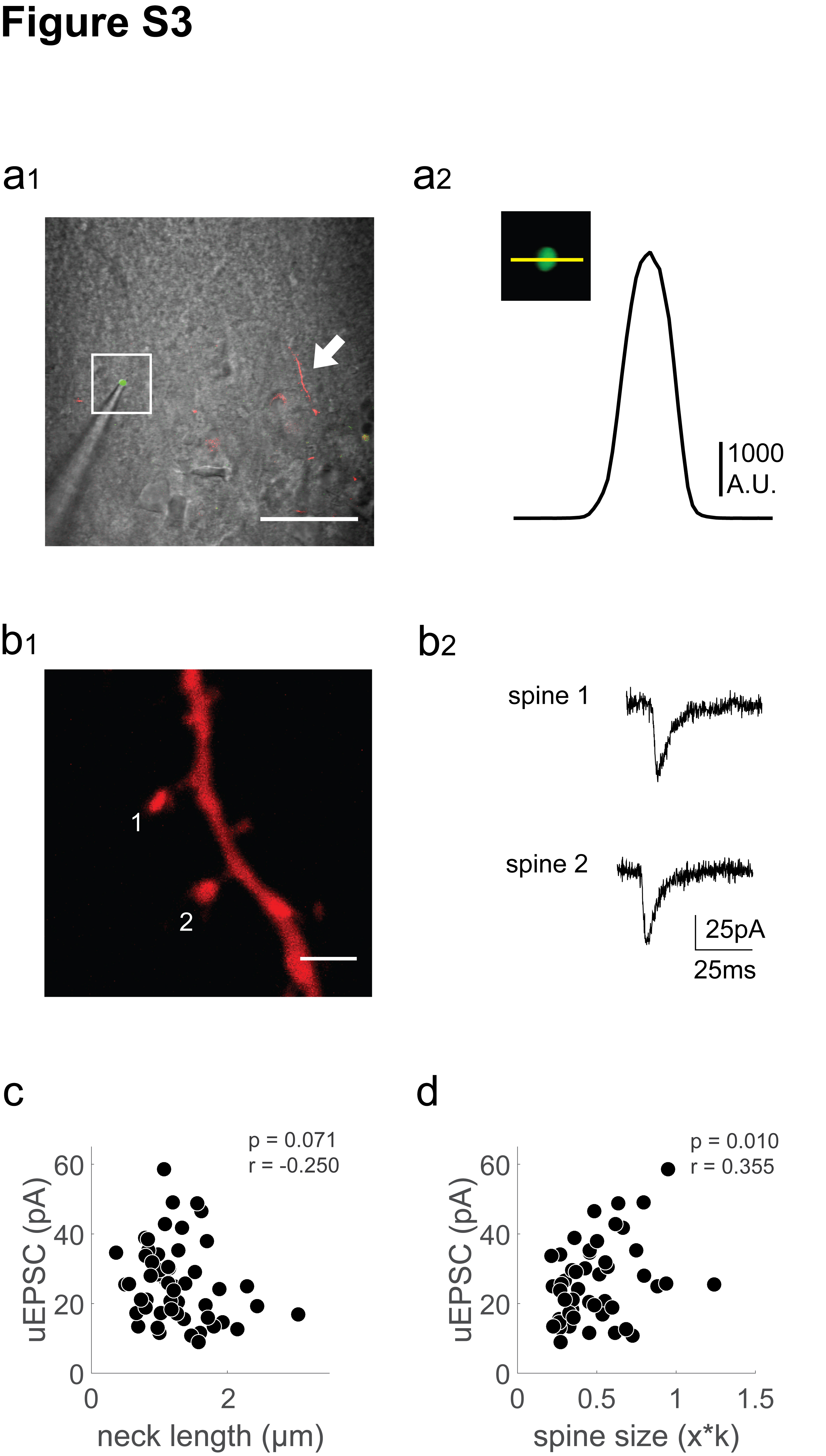

Supplement: FIGURE S3 — Calibration of uncaging laser intensity to compensate for interslice variability. (A1) Image of the target dendritic segment (white arrow) and the InSpeck microsphere in close proximity (white square). Scale bar: 50 μm. (A2) Intensity measurement of the bead (light intensity measured in A.U.). Inset: magnification of the bead and illustration of the line for determination of the intensity (yellow line). (B1) Z-projection of an imaged dendritic segment. Scale bar: 2 μm. (B2) uEPSCs corresponding to the spines marked in (B1). (C) Correlation of neck length and uEPSC. (D) Correlation of spine size and uEPSC. Statistics: spearman correlation. (D) Correlation of spine size and uEPSC. Statistics: spearman correlation. [file Image_3.JPEG]

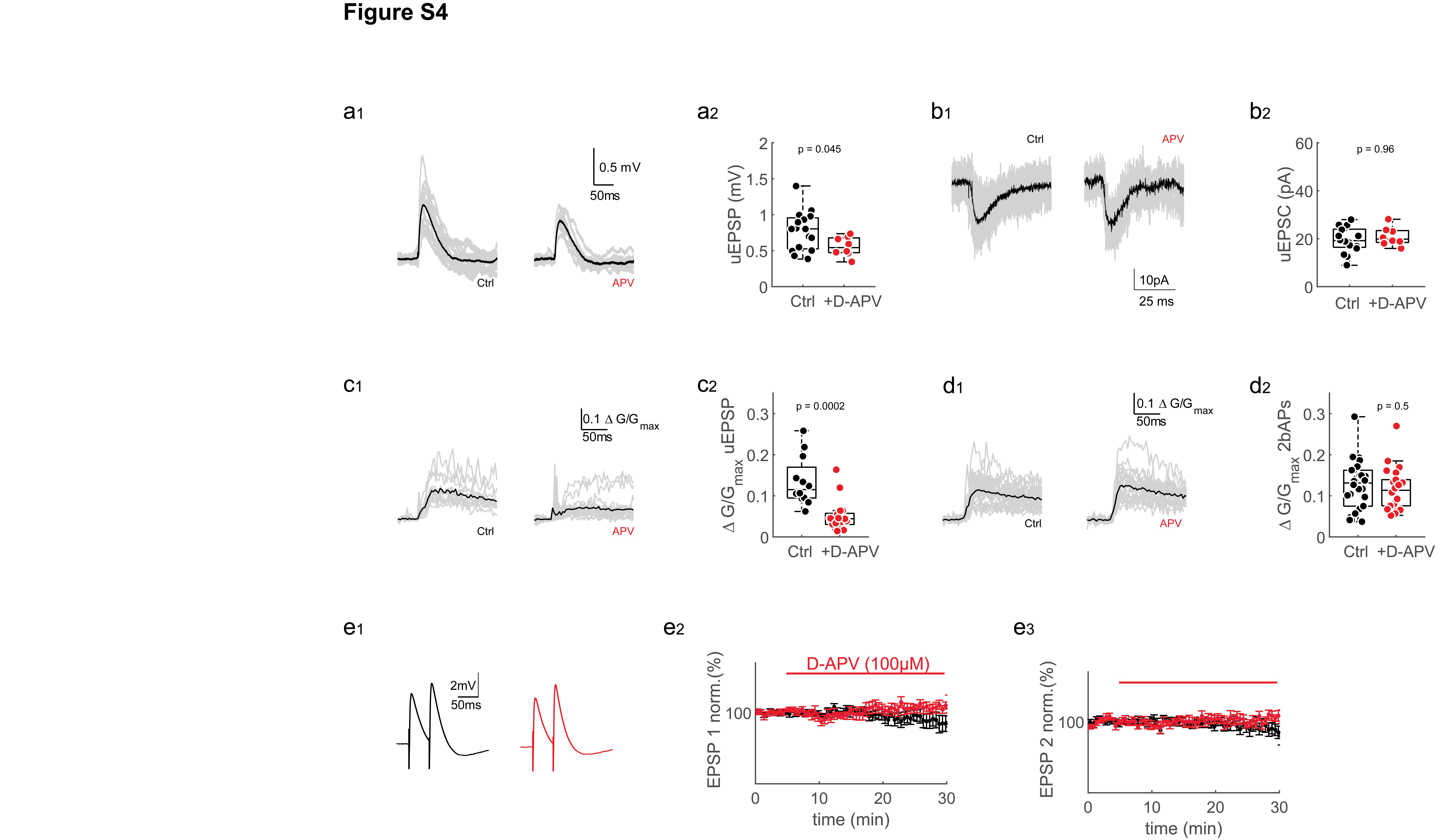

Supplement: FIGURE S4 — Activation of NMDARs by two-photon uncaging but not by electrical synaptic stimulation. (A1) Uncaging evoked EPSPs from different spines under control conditions (left panel, mean is depicted in black) and preincubated in D-APV (right panel, mean is depicted in black). (A2) Median boxplot of uncaging evoked uEPSPs under control conditions and in D-APV. Statistics: Wilcoxon-Mann-Whitney Test. (B1) Uncaging evoked EPSCs under control conditions (left panel, mean is depicted in black) and in D-APV (right panel, mean is depicted in black). (B2) Median boxplot of uncaging evoked uEPSCs under control conditions and in D-APV. Statistics: Wilcoxon-Mann-Whitney Test. (C1) Uncaging evoked Ca2+ transients under control conditions (left panel, mean is depicted in black) and in D-APV (right panel, mean is depicted in black) from the same spines as in (A). (C2) Median boxplot of the uncaging evoked Ca2+ transients depicted in (C1). Statistics: Wilcoxon-Mann-Whitney Test. (D1) bAP-doublet evoked Ca2+ transients under control conditions (left panel, mean is depicted in black) and in D-APV (right panel, mean is depicted in black). (D2) Median boxplot of bAP-doublet evoked Ca2+ transients under control conditions and in D-APV. Statistics: Wilcoxon-Mann-Whitney Test. (E1) Paired-pulse extracellular synaptic stimulation evoked compound EPSPs under control conditions (black) and 20–25 min after wash-in of 100 μM D-APV (red). (E2,E3) Time plots of normalized, binned (1 min) maximum amplitudes of EPSP1 (E2) and EPSP2 (E3) under control conditions (black) and during wash-in of D-APV (red). [file Image_4.JPEG]
